# Supramolecular Host–Guest Hydrogels for Corneal Regeneration

**DOI:** 10.3390/gels7040163

**Published:** 2021-10-05

**Authors:** Amy C. Madl, David Myung

**Affiliations:** 1 Department of Chemical Engineering, Stanford University, Stanford, CA 94305, USA; amadl@stanford.edu; 2 Byers Eye Institute, Stanford University School of Medicine, Palo Alto, CA 94303, USA; 3 VA Palo Alto Health Care System, Palo Alto, CA 94304, USA

**Keywords:** supramolecular hydrogels, corneal regeneration, host–guest chemistry

## Abstract

Over 6.2 million people worldwide suffer from moderate to severe vision loss due to corneal disease. While transplantation with allogenic donor tissue is sight-restoring for many patients with corneal blindness, this treatment modality is limited by long waiting lists and high rejection rates, particularly in patients with severe tissue damage and ocular surface pathologies. Hydrogel biomaterials represent a promising alternative to donor tissue for scalable, nonimmunogenic corneal reconstruction. However, implanted hydrogel materials require invasive surgeries and do not precisely conform to tissue defects, increasing the risk of patient discomfort, infection, and visual distortions. Moreover, most hydrogel crosslinking chemistries for the in situ formation of hydrogels exhibit off-target effects such as cross-reactivity with biological structures and/or result in extractable solutes that can have an impact on wound-healing and inflammation. To address the need for cytocompatible, minimally invasive, injectable tissue substitutes, host–guest interactions have emerged as an important crosslinking strategy. This review provides an overview of host–guest hydrogels as injectable therapeutics and highlights the potential application of host–guest interactions in the design of corneal stromal tissue substitutes.

## 1. Introduction

**Figure 1:**
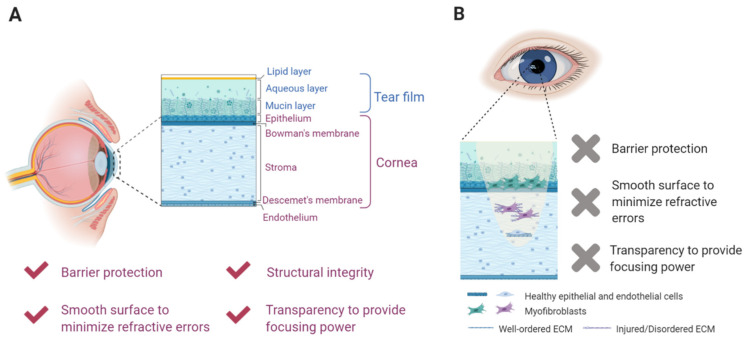
Corneal damage commonly results in visual disturbances. (**A**) The multilayered cornea provides barrier protection, promotes structural integrity, and provides focusing power for the visual system. (**B**) Trauma or disease can alter the structure of the cornea, resulting in impaired vision.

The cornea is a transparent optical interface that is responsible for two-thirds of the eye’s refractive power. The cornea also protects intraocular structures from the external environment and maintains the eye’s structural integrity (Figure 1A). Damage to this complex, multilayered tissue is sight-threatening, with an estimated 6.2 million people worldwide impacted by cornea-related vision loss and over 250 million others suffering from visual impairment due to corneal damage (Figure 1B) [1,2,3,4]. Moreover, Jeng and Ahmad have recently reported that 12.7 million people worldwide have moderate to severe vision loss (vision less than 20/60) amenable to surgical correction and are actively awaiting a corneal transplant [5]. The outermost layer of the cornea—the corneal epithelium—is ordinarily self-renewing, with adult stem cells from the surrounding limbus repopulating the epithelium after typical turnover and minor trauma [6]. Nevertheless, vision-compromising corneal pathologies can arise following superficial injuries when the limbal stem cell population is deficient due to disease or trauma (e.g., ocular burns) [3,7]. 

**Figure 2:**
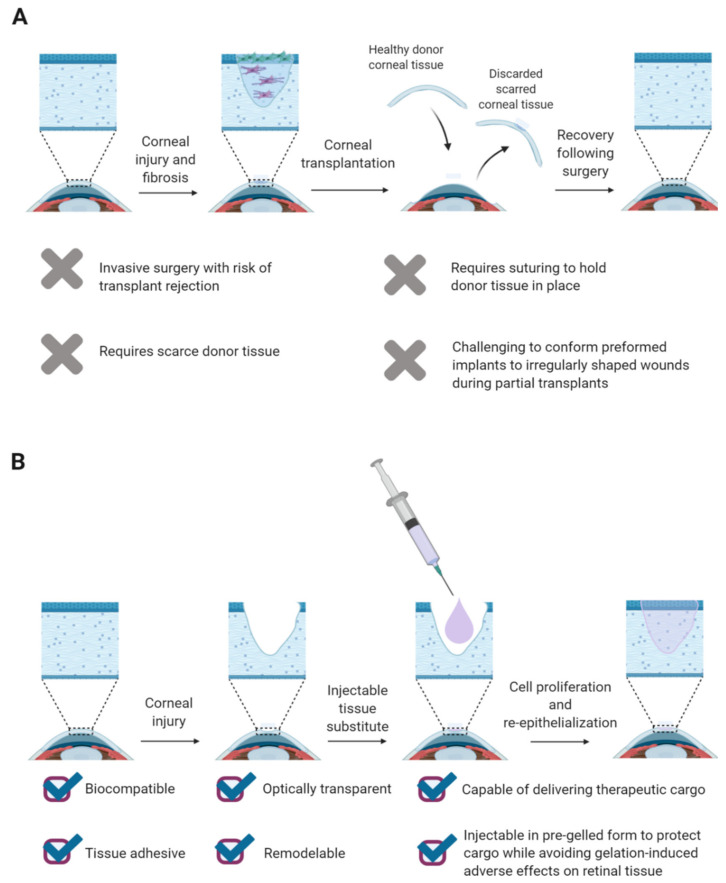
Injectable hydrogels are a promising alternative to allogenic donor tissue for corneal reconstruction surgeries. (**A**) Corneal transplantation is the gold standard for patients suffering from corneal blindness, but the therapeutic modality suffers from several limitations. (**B**) Injectable hydrogels that satisfy certain design criteria are promising alternatives to allogenic donor tissue in corneal reconstruction surgeries.

In patients with superficial corneal abrasions, epithelial healing typically proceeds without scarring [3]. However, permanent scarring and opacification often follow deeper injuries to the highly organized corneal stroma, which comprises about 90% of the cornea’s thickness and maintains corneal curvature, mechanical strength, and transparency [3,7,8]. Corneal transplantation is the gold standard treatment for patients with stromal tissue damage (Figure 2A) [7]. The cornea is the most transplanted tissue in the United States, with about 40,000 corneal transplants performed annually [3]. However, this treatment modality is hindered by long waiting lists, especially in the developing world. A recent study estimates that only one donor cornea is available for every 70 needed worldwide [9]. Moreover, patient outcomes are mixed following implantation of allogenic donor tissue, despite the cornea’s status as a relatively immune-privileged tissue that makes it one of the most successfully transplanted tissues. The modern transition away from traditional penetrating keratoplasty, in which a full rather than a partial thickness corneal tissue is transplanted, has increased success rates, but 10% of donor corneas are still rejected within the first year of transplantation, with a heightened rejection risk observed in patients with ocular burns, corneal nerve damage, or severe ocular surface pathologies (e.g., Stevens-Johnson syndrome and Sjögren’s syndrome) [3,4,10]. 

Plastic-based artificial corneas (keratoprostheses) serve as an alternative to donor tissue for corneal replacements, restoring minimal light transmission and ocular surface barrier protection [4]. However, these medical devices are associated with complications ranging from retinal detachment to glaucoma and are employed as a last resort in complex corneal blindness cases [10]. Hydrogels—scaffolds formed from water-swollen polymer networks—are a promising engineered alternative to keratoprostheses and donor tissue for scalable, nonimmunogenic corneal tissue repair. Specifically, injectable hydrogels are highly desirable for ophthalmic tissue engineering because they can be introduced in a minimally invasive manner to precisely fill tissue defects and homogeneously distribute encapsulated cargo (Figure 2B). 

Injectable hydrogels can be divided into two major classes: (i) materials that chemically crosslink to form gels in situ, which are injected into stromal defects as viscous precursor solutions and transition into gels over time; (ii) shear-thinning, self-healing hydrogels (e.g., host–guest hydrogels) that exist as gels within a syringe, flow through the needle under applied stress, and rapidly reform post-extrusion [11,12]. Both material classes exhibit advantageous properties that can be exploited in the design of corneal defect fillers. Illustratively, in situ gelling platforms promote tight wound apposition, and the physical and chemical properties of these materials can be tuned to recapitulate important features of the native corneal extracellular matrix, directing tissue repair and promoting functional recovery [13,14]. However, many in situ forming gels rely on crosslinking chemistries that may interfere with native biomacromolecules or cells and yield brittle hydrogel networks. In contrast, shear-thinning, self-healing hydrogels recapitulate the dynamic properties of native tissue and provide protection to encapsulated payloads during the injection process. Additionally, shear-thinning, self-healing hydrogels arise from selective, non-covalent interactions rather than covalent crosslinks, reducing cytotoxicity concerns but also resulting in weaker, less tunable mechanical properties. Both in situ forming and shear-thinning, self-healing hydrogels can be employed as acellular matrices to stabilize deep wounds, as well as cell-encapsulating gels that deliver differentiated cells or adult stem cells to the defect to more actively promote wound-healing and tissue remodeling.

In this review, we discuss the potential of host–guest hydrogels, an emerging class of injectable, shear-thinning, self-healing biomaterials that has not yet been exploited in ophthalmic regeneration applications, as a corneal stromal tissue substitute. We also explore how existing host–guest hydrogel systems can be adapted to recapitulate the unique strength and organization of corneal tissue to enable vision restoration following ocular trauma or disease.

## 2. Design Considerations for Corneal Stromal Substitutes

Reconstruction of the cornea presents significant engineering challenges due to its complex, multilayered structure. Among the five major layers of the cornea, three contain cells—the corneal epithelium, stroma, and corneal endothelium—and two, Bowman’s layer and Descemet’s membrane, are acellular collagenous interfaces [7]. The corneal epithelium, which ranges from five to seven cell layers, serves as a lubricating, protective barrier, minimizing friction during blinking and inhibiting intraocular damage due to pathogens and particulate matter [8]. Following injury, the epithelial regeneration that restores the functional ocular surface barrier is critical to reducing post-operative complications. Furthermore, corneal tissue, including the corneal epithelium, is highly innervated, with corneal nerves acting as homeostatic mechanical and thermal sensors [15,16,17]. To promote ocular health and vision restoration, engineered tissue substitutes should support corneal reinnervation in patients whose corneal nerves have been damaged due to trauma or disease.

In addition to enabling corneal reepithelialization and reinnervation, engineered materials for cornea reconstruction must mimic the stabilizing function of the healthy corneal stroma. The native cornea is subjected to sustained load due to intraocular pressure from the fluid-filled, pressurized globe [18]. As a result, the native cornea is viscoelastic and exhibits nonlinear mechanical properties, with an elastic modulus that increases under increasing loads [18]. Ideal tissue substitutes would recapitulate these viscoelastic properties while providing a biomimetic microenvironment that supports endogenous cells and facilitates the regeneration of multilayered corneal tissue. 

The stroma underlying the corneal epithelium and Bowman’s layer comprises about 90% of the cornea’s thickness and consists of organized layers of aligned collagen fibrils consisting of types I, V, and VI collagen [3,7]. The corneal stroma is collagen-rich, with collagen fibrils exhibiting feature sizes that are significantly smaller than visible spectrum wavelengths. Illustratively, collagen fibril diameters in the stroma range from 22.5 to 35 nm with a mean interfibrillar spacing of 41.5 nm [7]. Parallel collagen fibrils in the stroma are precisely arranged into 200–300 layers called lamellae [18]. Each lamellar sheet is oriented in a direction orthogonal to each adjacent lamella, an organization that maintains corneal curvature and improves mechanical strength and resilience. In addition to collagen, the stroma contains proteoglycans and glycosaminoglycans (GAGs), such as keratan sulfate, decorin, lumican, and keratocan, that maintain collagen interfibrillar spacing and corneal hydration [3]. Keratan sulfate is the most abundant GAG in the corneal stroma, accounting for 65% of stromal GAGs. 

While the cellular density of the cornea is relatively low, the stromal keratocytes of mesenchymal origin play an important role in the visual system by secreting and maintaining the organized extracellular matrix. These mitotically quiescent dendritic cells occupy only from 3 to 5% of stromal volume and contact each other via elongated cytoplasmic processes [7]. Moreover, the cells of the single-layered corneal endothelium maintain tissue transparency by regulating hydration [6,8]. Although there is evidence suggesting that the endothelial layer can heal to a limited extent, for instance, in reported cases of Descemetorhexis Without Endothelial Keratoplasty (DWEK), endothelial cells themselves do not readily regenerate the way epithelial cells do, posing a significant barrier to functional recovery following deep tissue injury [19].

To restore visual acuity after corneal damage, engineered tissue substitutes must be biocompatible, optically transparent, and mechanically robust. Furthermore, engineered materials for corneal reconstruction should be tissue-adhesive, limit the need for invasive sutures, and be remodelable and capable of delivering therapeutic cargo (e.g., antibiotics or regeneration-promoting growth factors such as nerve growth factor). In addition, corneal tissue substitutes should either be precisely cut to match or be capable of conforming to defect site geometry, presenting a smooth surface with minimal disparities in size, thickness, and curvature between the replacement and recipient tissue. As the corneal shape dictates most of the eye’s refractive power and is thus responsible for visual acuity, poor wound apposition and surface distortions due to an imperfectly fitted tissue replacement may induce astigmatism [18]. 

## 3. Current Hydrogel-Based Approaches to Corneal Regeneration

Hydrogels prepared from reconstituted type I collagen are commonly employed in ophthalmic tissue engineering due to the high native type I collagen content in the eye [3,20,21], with recently reported systems harnessing biorthogonal crosslinking chemistries to improve the mechanical properties of the reconstituted extracellular matrix (ECM) [22,23,24]. Other commonly used biopolymers for corneal regeneration include gelatin, silk, dextran, and hyaluronic acid. Moreover, human and animal stromal tissues that were decellularized using non-ionic detergents or enzymes have been employed as tissue substitutes [4]. While these scaffolds are promising, they pose a disease transmission risk and cannot be readily tailored to fit wounds of any size or shape. 

To improve scalability and reduce immunogenic concerns, synthetic hydrogels, including poly(ethylene glycol)-based materials, are also under investigation for corneal reconstruction [10]. Current hydrogel-based alternatives to corneal transplantation have been comprehensively summarized, and interested readers are directed to several excellent reviews on the topic [3,7,18,21]. To highlight the diverse approaches adopted by researchers tackling corneal regeneration, four recently published systems are discussed below, along with potential translation pitfalls associated with these engineered systems.

**Figure 3:**
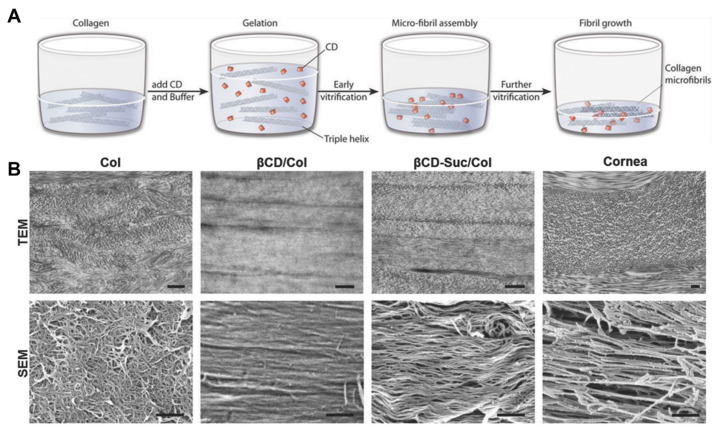
Cyclodextrins modulate collagen assembly during vitrification, resulting in biomimetic fibril organization. (**A**) Collagen vitrigels prepared by mixing equal volumes of collagen and CD-buffered solution. (**B**) Transmission electron microscopy (TEM) and scanning electron microscopy (SEM) of Col, βCD, and succinyl-functionalized βCD (βCD-Suc), and native rabbit cornea, to demonstrate effect of CD functionality on collagen ultrastructure organization. Scale bar = 500 nm. Reproduced with permission. Copyright 2018, Wiley-VCH [25].

In 2018, Singh, Elisseeff, and coworkers reported cyclodextrin modulated type I collagen assembly during vitrification, which produced mechanically robust, transparent βCD/Col gels with aligned fibers and lamellae (Figure 3) [25]. During gelation, cyclodextrins play a role similar to small leucine-rich proteoglycans in corneal development, regulating fibril diameter and spacing through interactions with hydrophobic amino acids in collagen to yield highly organized, biomimetic microstructures. Acellular, curved βCD/Col hydrogel implants prepared using custom molds successfully integrated with tissue in a rabbit partial keratoplasty model and supported re-epithelialization in the ex vivo rabbit model. However, the platform’s potential is limited by its preparation process, which does not readily permit custom fitted materials for irregularly sized cavities and relies on pH and temperature conditions that may denature encapsulated payloads.

Embracing a different strategy, Annabi, Dana, and colleagues created a visible light crosslinkable gelatin–methacryloyl bioadhesive for corneal reconstruction (GelCORE) [26]. This engineered system exhibited tunable mechanical properties based on polymer concentration and crosslinking time and could be photopolymerized in situ to precisely fit the defect geometry. Moreover, the hydrogel system promoted stromal regeneration and re-epithelialization in a 50% thickness rabbit stromal defect model. 

An alternative, light-free in situ forming adhesive was reported by Griffith and coworkers in 2020 [27]. This hydrogel platform (LiQD Cornea) does not employ UV or visible light exposure to trigger gelation. Instead, the corneal stromal tissue substitute relies on fibrinogen and 8-arm polyethylene glycol modified with collagen like peptides to form a porous hydrogel within 5 min of mixing with thrombin and the amide coupling reagent 4-(4,6-dimethoxy-1,3,5-triazin-2-yl)-4-methylmorpholinium chloride at physiological temperature. LiQD Cornea effectively sealed full-thickness corneal perforations in rabbits and facilitated regeneration of the corneal epithelium, stroma, and nerves in a 12-month pig study. 

In contrast to relatively non-specific chemistries such as free radical and amide coupling, bioorthogonal crosslinking reduces the gel-related risks to encapsulated payloads such as cells and growth factors, and to the underlying wounded tissue the hydrogel is designed to fill and treat [28]. Illustratively, copper-free, SPAAC-based click chemistry facilitates rapid gelation under physiological conditions without relying on light energy or chemical catalysts to initiate covalent crosslinking. Demonstrating the power of this approach in ophthalmic regeneration applications, our lab recently reported several differently bioorthogonally crosslinked, in situ forming corneal tissue substitutes that fill and stabilize deep corneal wounds in vivo, promoting multi-layered epithelialization and tight junction formation while presenting biomimetic optical properties [22,23,24].

## 4. Potential of Host–Guest Hydrogels in Ophthalmic Tissue Engineering

Preformed materials require invasive surgical procedures to implant, increasing infection risk and patient discomfort [12]. Accordingly, injectable materials such as those described above are highly desirable for corneal tissue engineering because they can be introduced in a minimally invasive manner to precisely fill a defect. Injectable hydrogels can be in situ forming or shear-thinning, self-healing gels. In situ gelling systems exist as a liquid in a syringe and form a hydrogel network after extrusion into the wound site. Gelation times for in situ forming hydrogels must be matched to the desired application to avoid material loss in the defect and to prevent premature gelation, which can cause clogging challenges in rapidly gelling systems [29]. Common crosslinking chemistries for in situ forming hydrogels include radical initiation and Michael addition crosslinking, which may denature encapsulated protein therapeutics and present cytotoxicity concerns due to off-target reactivity with biological structures.

In contrast, preformed supramolecular gels with shear-thinning and self-healing properties may be extruded via shear-induced flow [29]. Upon application of shear stress, the viscosity of shear-thinning hydrogels is reduced, enabling flow through a syringe needle [11]. Rapid bond reformation when the stress is removed enables material retention after injection into a defect. Gel formation prior to injection can result in increased payload homogeneity, higher post-injection cell viability, and reduced denaturation of encapsulated cargo [11,30], making shear-thinning and self-healing hydrogels highly desirable for sensitive ocular tissue engineering applications. The shear-thinning properties of supramolecular hydrogels also make them desirable materials for 3D printing applications, which have become increasingly popular in corneal tissue engineering [31,32,33].

In addition to aiding material placement, the dynamic bonds in shear-thinning and self-healing supramolecular hydrogels recapitulate native tissue properties. Biological materials are commonly formed via non-covalent interactions and exhibit time-dependent (viscoelastic) mechanical properties. Specifically, nature relies on electrostatic interactions, hydrogen bonding, van der Waals forces, π–π interactions, and hydrophobic interactions to drive molecular assembly into complex structures [30,34]. These dynamic linkages enable cell-mediated matrix reorganization and can promote proliferation and differentiation in several cell lines [35,36]. In contrast, covalent chemical crosslinking, which is commonly employed in implanted and in situ forming hydrogel biomaterials, generally yields brittle scaffolds with poor transparency properties [37]. Although cell-mediated remodeling of covalently crosslinked materials can occur through enzymatic degradation of the matrix, this approach requires careful consideration of cell enzyme secretion over space and time.

**Figure 4:**
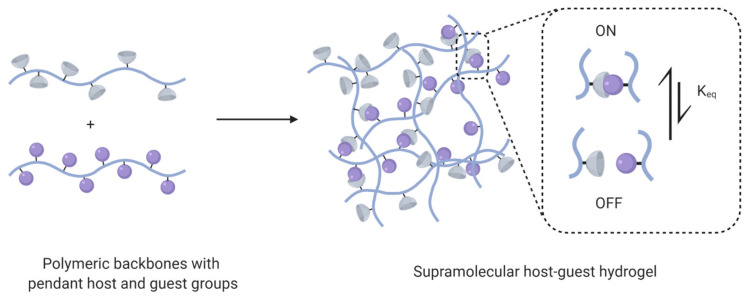
A host–guest hydrogel forms upon mixing of polymers modified with pendant host and guest groups due to the reversible formation of inclusion complexes.

Host–guest interactions are particularly popular in supramolecular hydrogel design due to their tunability and specificity [30,38]. In host–guest hydrogels, transient interactions between molecules containing a cavity and complementary guest groups form a physical network. These interactions are reasonably specific, with the inclusion of an appropriately sized guest group in the host macrocycle minimizing polar solvent interactions. As these systems do not rely on non-specific interactions or long-range order development for network formation, host–guest hydrogels quickly assemble upon mixing and rapidly recover following shear thinning [29]. In host–guest systems, polymers are typically modified with guest and host groups, either as terminal or pendant moieties (Figure 4). Although guest and host groups may be present on the same backbone, this presentation results in loop formation and intrachain junctions and is generally avoided [39]. 

A broad array of interaction strengths is available in host–guest hydrogels through judicious choice of host–guest pairs (Table 1). In hydrogel networks, the associative equilibrium constant K_eq_ describes the degree of network association, while the associative (k_a_) and dissociative (k_d_) rate constants are associated with network binding dynamics [37]. For a given macrocyclic host, K_eq_ can be altered by varying the size and properties of the guest group. In addition, gel strength and shear-thinning and self-healing properties can be tuned by varying the concentration of both host and guest groups, as well as the valency of the crosslinked groups [37,40]. Furthermore, host groups can be used for drug and growth factor sequestration when non-stoichiometric amounts of host and guest groups are presented in the hydrogel network [30]. The inclusion of therapeutics in grafted macrocycles enables sustained small-molecule release, a promising feature for corneal tissue regeneration applications since topically applied drugs are rapidly cleared during blink cycles. Moreover, in hydrogels with excess host groups, host–guest complexation of guest-bound peptide sequences can be used to dynamically control biomolecule display [41,42]. However, this dynamic display approach may be better suited to primarily covalent systems where the complexation of bioactive signal does not compete with crosslinking interactions, potentially compromising hydrogel integrity over time.

**Figure 5:**
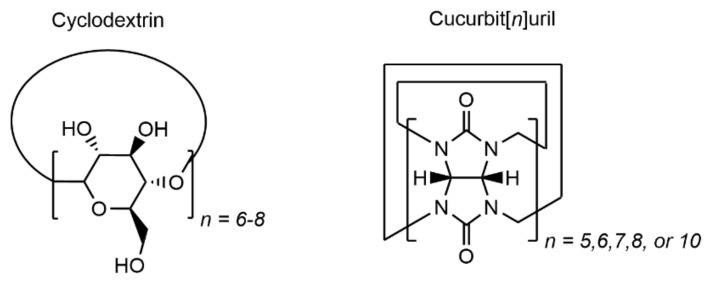
Chemical structures of the common host molecules cyclodextrins and cucurbit[*n*]urils. For α-cyclodextrin, *n* = 6, for β-cyclodextrin, *n* = 7, and for γ-cyclodextrin, *n* = 8.

Cyclodextrin and cucurbit[*n*]uril hosts (Figure 5) are commonly employed in host–guest hydrogel biomaterials due to their aqueous solubility and biocompatibility [43,44]. The use of other macrocyclic host groups like crown ethers, catenanes, and porphyrins in hydrogels is solubility-limited [37]. However, the range of water-soluble macrocyclic hosts is expanding along with interest in host–guest interactions, with Xue, Zavalij, and Issacs recently reporting a new family of pillar[*n*]arene sulfate hosts with high affinity for quaternary diammonium ions [45]. 

### 4.1. Cyclodextrin-Based Hydrogels

Cyclodextrins (CDs) are seminatural products composed of α-D-glucopyranoside units cyclically coupled through α-1-4-glycosidic linkages to yield a 3D truncated cone with a hydroxyl-rich hydrophilic surface and a relatively hydrophobic interior [30,51]. α-, β-, and γ-cyclodextrins are composed of 6, 7, and 8 glucopyranose units, respectively. Cyclodextrins are broadly used as excipients and solubilizing agents in pharmaceutical applications and are “generally recognized as safe” by the U.S. Food and Drug Administration [30,34,51]. Cyclodextrin–guest interactions are driven by hydrophobic and van der Waals interactions between the hydrophobic cavity and the guest molecule, as well as hydrogen-bonding interactions between the guest and the cyclodextrin surface. Guest molecules less polar than water readily displace energetically unfavorable water molecules in the cyclodextrin cavity [51], while hydrogen bonding between neighboring cyclodextrin groups can promote self-association [38].

Cyclodextrins preferentially bind neutral and anionic guests [38], displaying strong affinity for a wide variety of small-molecule guests [52]. Due to their differing cavity sizes, different cyclodextrins act as hosts for different hydrophobic groups, with α-CD binding linear alkyl chains and β-CD associating with diamondoid guests such as adamantine. Stimuli-responsive guests such as azobenzene and ferrocene are also employed in CD systems [30,53]. The associative equilibrium constant for cyclodextrin–guest interactions range from 10 M^−1^ to 10^5^ M^−1^, with the commonly employed β-CD–adamantane interaction exhibiting a K_eq_ between 10^4^ and 10^5^ M^−1^.

In 2013, Rodell, Kaminski, and Burdick reported a shear-thinning hyaluronic acid hydrogel composed of adamantane modified hyaluronic acid (AD-HA) and β-cyclodextrin modified HA (CD-HA) [54]. Upon component mixing, a hydrogel with host–guest bonds was rapidly formed, with physical properties dependent on macromer concentration, extent of guest modification, and the molar ratio of host and guest groups. In later work, Rosales et al. demonstrated that stiffness in supramolecular hyaluronic acid-based hydrogels could be modulated with light using azobenzene guest groups, which exhibit higher affinity for β-cyclodextrin in the *trans* state [55]. However, these host–guest hyaluronic acid hydrogels are not suitable for long-term cell culture applications as they are rapidly diluted in cell culture medium [56,57].

More recently, dynamic hydrogels with tunable mechanical properties have been prepared via thiol-allylether photo-click reactions using thiolated poly(vinyl alcohol) (TPVA), 4-arm poly(ethylene glycol)-allylether (PEG4AE), and mono-functional β-cyclodextrin-allylether (βCDAE) [58]. The addition of adamantane-functionalized 4-arm PEG stiffens the network through host–guest interactions with pendant β-cyclodextrin groups; these interactions can be reversed by adding unmodified β-cyclodextrin that competes for the adamantane guest groups. Similarly, Sisso, Boit, and DeForest exploited interactions between β-cyclodextrin and adamantane on modified gelatins (Gel-CD and Gel-AD, respectively) to prepare self-healing, injectable hydrogels with cell-adhesive properties [59]. Gel-CD and Gel-AD flow at room temperature due to their extensive modification with bulky pendant groups, but mixing Gel-CD and Gel-AD at high weight percentages (40% *w/v* total) yielded soft hydrogels with storage moduli up to approximately 400 Pa.

In addition to host–guest gelatin hydrogels prepared using pendant host and guest groups, supramolecular gelatin hydrogels were fabricated by precomplexing aromatic residues in gelatin with freely diffusing acrylated β-cyclodextrin monomers [60,61,62]. Post-photocrosslinking, the mechanically robust hydrogels are moldable and injectable, with excess Ac-β-CD monomers facilitating bioadhesiveness and hydrophobic drug delivery. In a follow-up work, the authors improved the in vivo stability of their platform by incorporating a small number of chemical crosslinks using diacrylated monomers [61]. While the additional covalent crosslinks improved retention, the reversible host–guest crosslinks enabled injectability in the pre-gelled state, sustained hydrophobic drug release, and cellular infiltration.

The sustained delivery of small molecule therapeutics from supramolecular AD-HA and CD-HA hydrogels has also been demonstrated [52]. Most hydrogels exhibit large mesh sizes, resulting in the rapid diffusion of encapsulated small molecules. To achieve a sustained release profile for hydrophobic drugs, excess cavitands can be employed as therapeutic depots, with release kinetics tuned by varying cyclodextrin content or drug-cyclodextrin affinity [52,63]. However, this approach may not be generalizable across polymeric backbones, as excess cyclodextrin groups inhibited gel formation in the recently reported Gel-CD/Gel-AD system [59].

Host–guest interactions have been underemployed in ophthalmic regeneration applications to date, with cyclodextrins primarily used as drug depots in covalently crosslinked hydrogels. For example, ocular delivery of the antimicrobial 5,6-dimethoxy-1-indanone N4-allyl thiosemicarbazone (TSC) using β-cyclodextrin functionalized hydrogels was reported in 2013 [64]. Employing this system, Glisoni et al. reported controlled TSC release for at least two weeks at concentrations within the optical therapeutic window for ocular treatment. Similarly, poly(2-hydroxyethyl methacrylate) contact lens hydrogels modified with β-cyclodextrin (pHEMA/β-CD) have been shown to deliver the anti-glaucoma drug puerarin though the cornea, resulting in a higher aqueous humor viability than topically applied puerarin eye drops [65].

Additionally, cyclodextrin-based poly(pseudo)rotaxanes have been used to fabricate supramolecular hydrogels for ocular applications. Unlike traditional host–guest hydrogels, in which interactions between host macrocycles and guest molecules create crosslinks between polymer chains, cyclodextrin-based poly(pseudo)rotaxane hydrogels are formed through hydrogen bonding interactions between cyclodextrins threaded on different linear polymer chains [39]. Illustratively, Zhang et al. prepared a micellar hydrogel for topical ocular drug delivery using low-molecular-weight monomethoxy-PEG-PCL polymer micelles and α-CD, which was shown not to irritate rabbit eyes in a modified Draize test [66]. This micellar hydrogel system has been used to co-encapsulate and co-deliver dexamethasone sodium phosphate and Avastin in a rat alkali burn model, resulting in reduced corneal inflammation and neovascularization [67].

While we are not aware of any publications applying traditional host–guest hydrogels for corneal regeneration, preliminary work from our group suggests that encapsulation of human corneal stromal stem cells in supramolecular host–guest hyaluronic acid hydrogels promotes high cell viability in keratectomy wounds and enhanced re-epithelialization in organ culture [68]. Moreover, initial experiments indicate that this supramolecular gel system supports epithelial cell delivery following complete epithelial debridement of the cornea [69].

### 4.2. Cucurbit[n]uril-Based Hydrogels

Cucurbit[*n*]urils (CB[*n*]s) are rigid, pumpkin-shaped cavitands containing repeating *n* glycouril units linked by methylene bridges. CB[*n*]s possess hydrophobic cavities lined with polar carbonyl groups [70,71,72]. Guest interactions with CB[*n*]s are similar to cyclodextrin–guest interactions, although the driving force is stronger due the release of high-energy water molecules from the CB[n] cavity upon guest inclusion. Additionally, ion–dipole interactions with carbonyl groups play a significant role in guest inclusion, with CB[*n*] preferentially binding cationic guests [38,70]. Uniquely, CB[8] can bind two guests simultaneously, forming homoternary or heteroternary complexes. As a result, CB[8]-based hydrogels can be prepared by mixing polymers with pendant guest groups with freely diffusing CB[8] [34]. When CB[8] associates with two different guest groups to form a heteroternary complex, the electron deficient guest binds CB[8] before the electron rich guest [37]. Charge transfer or π–π stacking interactions between guest groups further reinforce binding affinity in platforms employing CB[8]-based crosslinks [30].

CB[*n*]-guest binding is typically stronger than CD-guest binding. CB[6] associates with neutral and positively charged organic guests, such as 1,6-diaminohexane, and exhibits exceptionally high selectivity toward alkylammonium ions. CB[6]–guest interactions exhibit associative equilibrium constants up to 10^12^ M^−1^ and are typically about 10^5^ for alkyl ammonium salts [37,46,48]. CB[7] associates with larger amphiphilic guests with a broad range of binding affinities, with reported associative equilibrium constants as high as 10^17^ M^−1^ and typical K_eq_ values between 10^5^ and 10^9^ M^−1^ [37,48,73]. Similarly, typical CB[8]-guest associative equilibrium constants range from 10^4^ and 10^6^ for each guest [37,48]. CB[8] binds positively charged molecules, with aromatic amino acids, viologen, and 2-naphthoxy commonly employed as guest groups in CB[8]-based hydrogels.

In 2012, Hahn, Kim, and coauthors reported a biocompatible hydrogel system prepared using cucurbit[6]uril-conjugated hyaluronic acid (CB[6]-HA) and diaminohexane-conjugated HA (DAH-HA) [74]. Hydrogel assembly using a low-polymer-weight percentage (2 wt%) proceeded in the presence of cells, yielding soft but robust hydrogels with storage moduli around 2.4 kPa. In addition, in situ hydrogel formation occurred when CB[6]-HA and DAH-HA solutions were sequentially injected under the skin of nude mice, and the non-stoichiometric presentation of guest and host groups in the hydrogel enabled modular modification with CB[6]-functionalized molecules. In follow-up work, mesenchymal stromal cells encapsulated in CB[6]-based hyaluronic acid hydrogels remained viable in mice for more than 60 days post-injection [75].

**Figure 6:**
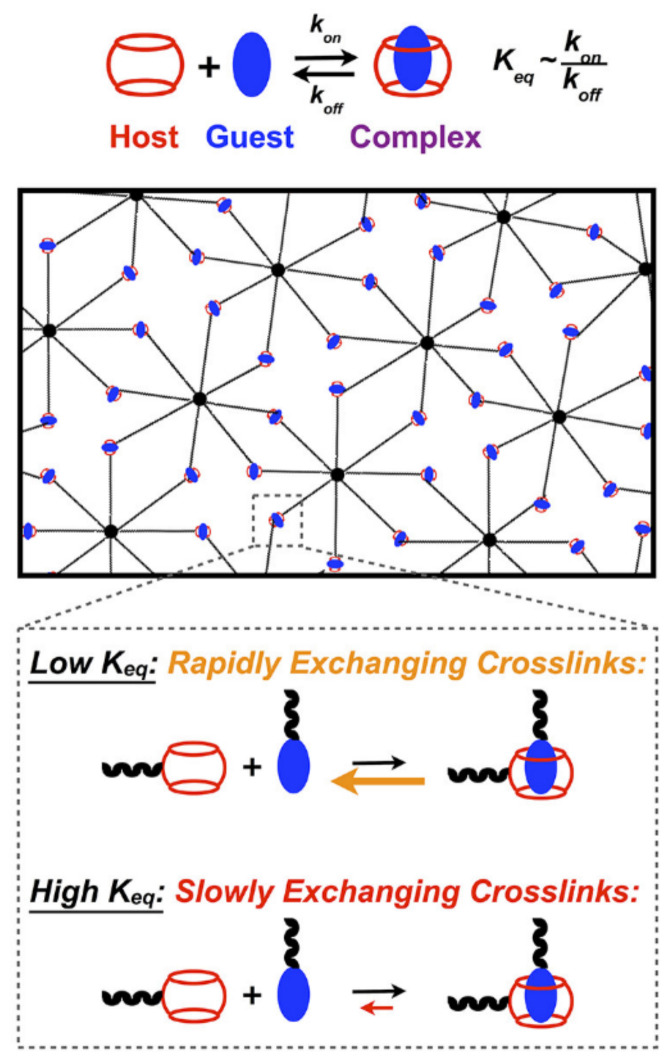
CB[7]-based hydrogels with a broad range of properties can be fabricated using guest groups with different affinities for CB[7]. Reproduced with permission. Copyright 2019, American Chemical Society [49].

CB[7]-based hydrogels have also been reported [49,76,77]. CB[7] binds a variety of guest groups with a broad range of host–guest affinities, enabling the facile preparation of hydrogels with tunable bulk properties. Illustratively, Zou, Braegelman, and Webber prepared host–guest hydrogels with divergent relaxation rates by mixing 8-arm PEGs with terminal CB[7] macrocycles with five different eight-arm PEGs with terminal guest groups of varying affinity (Figure 6) [49]. While each guest-modified macromer formed a hydrogel upon mixing with 8-arm PEG-CB[7], only guest groups with binding equilibrium constants less than 10^9^ M^-1^ resulted in gels with rapid self-healing and frequency-dependent mechanical properties; higher affinity guest group displayed frequency-independent storage moduli values reminiscent of covalent hydrogels and did not fully heal after cutting. Intriguingly, the wide range of CB[7]–guest affinities can be used to tune the gelation kinetics of CB[7]-based hydrogels by precomplexing CB[7] with competing guest groups, resulting in gelation times ranging from seconds to hours in CB[7]-adamantane gels [76].

Alternatively, Qiao et al. prepared CB[7]-based supramolecular hydrogels using unbound CB[7] [77]. The authors functionalized the cationic polysaccharide chitosan with catechol guest groups, which formed inclusion complexes with freely diffusing CB[7]. In turn, the CB[7] portal carbonyl groups bound γ-Fe2O3 nanoparticles through electrostatic interactions, creating a complex supramolecular network that enabled the magnetic regulation of thermo-chemotherapy.

Scherman and coworkers reported the first CB[8]-based hydrogel in 2010 [50]. In these hydrogels, crosslinks between polymer backbones were formed by supramolecular ternary complexes of freely diffusing CB[8] and pendant viologen and naphthoxy groups. The soft CB[8]-based hydrogels were highly colored due to the formation of charge-transfer complexes between the guest groups and exhibited tunable mesh sizes and mechanical properties (e.g., plateau moduli between 350 and 600 Pa). Appel et al. also prepared lightly colored, high-water-content CB[8]-based hydrogels using cellulosic derivatives modified with pendant viologen and naphthoxy groups [78]. Using different guest groups with varying CB[8] affinity, the Scherman group further prepared physically crosslinked hydrogels with the same network structure and strength but different network dynamics, facilitating the controlled in vitro release of the model drug Rhodamine-B [79].

In contrast with uncapped charged guest groups, which form heteroternary complexes with CB[8] and raise cytotoxicity concerns in in vivo applications [80], the aromatic amino acids phenylalanine and tryptophan can form 2:1 homoternary complexes with CB[8], enabling the fabrication of non-colored CB[8]-based hydrogels suitable for biomedical applications [81,82]. For example, injectable, tissue adhesive hydrogels prepared from CB[8] and hyaluronic acid functionalized with phenylalanine terminated peptides have shown promise as delivery vehicles in primary human glioma models [83].

Despite its promise, CB[8]’s poor aqueous solubility limits CB[8] loading and consequent mechanical properties for CB[8]-based hydrogel systems. To overcome CB[8]’s solubility limitations, the Scherman and Zhang groups have independently reported the photo-initiated in situ polymerization of CB[8] supramolecular hydrogel networks from monomer precursor solutions [84,85,86,87,88]. Building on this work, we recently reported the fabrication of CB[8]-based supramolecular gelatin hydrogels for cell encapsulation [89]. These optically transparent, shear thinning, injectable hydrogels form on demand via thiol–ene reactions between preassembled CB[8]·FGGC peptide ternary complexes and grafted norbornenes and promote encapsulated cell viability over at least seven days in culture.

As with cyclodextrin-mediated gels, network dynamics in CB[8]-based hydrogels can be modulated by incorporating stimuli-responsive guest groups. For example, the use of photodimerizable guest groups in CB[8]-based hydrogels enables on demand conversion of supramolecular crosslinks to covalent crosslinks [90]. When reversible photodimerizable groups such as *trans*-Brooker’s Merocyanine and coumarin are employed, partial switching between static and dynamic bonds is observed on certain polymer backbones, albeit under irradiation conditions that are unsuitable for cell culture [87,91].

## 5. Secondary Crosslinking for Improved Stability

Healthy corneal tissue is mechanically robust and exhibits depth-dependent strength [18]. Specifically, the anterior third of the cornea possesses a higher density of interlamellar collagen connecting fibers and exhibits increased tensile strength, which stabilizes surface curvature in the presence of intraocular pressure variations [18]. Commonly used physically crosslinked biopolymers in corneal tissue substitutes (e.g., type I collagen) do not recapitulate the mechanical properties of healthy tissue and are susceptible to cell-mediated remodeling and degradation, limiting their translation potential to promote corneal regeneration. To improve mechanical properties, biopolymers, including degraded collagen in damaged corneas, are often chemically crosslinked. Frequently employed crosslinkers include nonspecific bifunctional small molecules such as glutaraldehyde and 1-ethyl-3-(3-dimethyl aminopropyl) carbodiimide hydrochloride (EDC) that react with amine and/or carboxyl groups, resulting in poor bioselectivity that may limit clinical utility.

**Figure 7:**
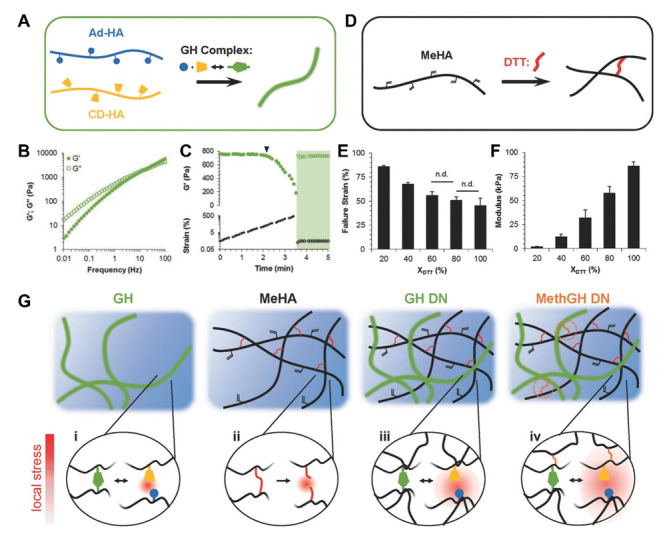
Dual network hyaluronic acid hydrogels exhibit enhanced resilience toward repeated loading. (**A**) Idealized schematic of adamantane (Ad-HA, blue) and β-cyclodextrin (CD-HA, yellow) modified hyaluronic acid crosslinked through host–guest (GH) complexation. (**B**,**C**) Frequency sweeps (**B**; 0.01–100 Hz, 0.5% strain) and strain sweeps (**C**; 1.0 Hz, 0.5–500% strain) for GH hydrogels (5.0 wt%) with yield point indicated (▾, 64% strain) and subsequent rapid recovery (shaded; 1.0 Hz, 0.5% strain). (**D**–**F**) Idealized schematic of Michael addition crosslinking (**D**) of methacrylated hyaluronic acid (MeHA) by DTT (red), where crosslink density was controlled through the thiol:methacrylate ratio (Χ DTT) and altered failure strains (**E**) and compressive elastic moduli (**F**). (*p* < 0.05, except where no difference (n.d.) is indicated). (**G**) Local stress under loading (red) dissipated through reversible GH complex rupture (**i**) within the primary GH network; increased stress led to covalent bond rupture (**ii**) within the secondary covalent network, while energy dissipation from the GH network reversibly protected the secondary MeHA network from bond rupture within the double networks (**iii**), a mechanism which was enhanced through network tethering to enable stress transference (**iv**). Reproduced with permission. Copyright 2016, Wiley-VCH [56].

Similarly, secondary networks have been introduced into supramolecular host–guest hydrogels to promote long-term stability and customize mechanical properties [56,92,93]. Host–guest hydrogels are typically weak and disassemble over time due to their spontaneously dissociating dynamic bonds, resulting in surface erosion that limits their utility in vitro studies [29,39,92]. Common secondary gelation mechanisms for host–guest hydrogels include photopolymerization and thermogelation [92]. Illustratively, the secondary covalent crosslinking of AD-HA and CD-HA hydrogels via the addition of thiols and Michael acceptors has been reported to increase hydrogel moduli and stability in vivo, with the presence of a dual network resulting in a 3.5-fold increase in stability in a myocardial infarct model at 28 days post-injection [92]. Likewise, dual network hydrogels composed of AD-HA, CD-HA, and MeHA with dithiothreitol exhibited enhanced resilience toward repeated loading while retaining the host–guest hydrogel’s rapid self-healing and injectability (Figure 7A–F) [56]. In dual networks containing methacrylate groups on the same HA backbone as host or guest molecules, the primary supramolecular network prevented internal rupture of covalent bonds under repetitive loading, maintaining network architecture and strength (Figure 7G).

In addition to promoting stability and resilience, a combination of host–guest crosslinks and covalent crosslinks can be used to modulate hydrogel viscoelasticity to better recapitulate the healthy tissue microenvironment. For example, Hui et al. reported modular hydrogels with light-mediated covalent and supramolecular interactions to match liver tissue mechanics [94]. Specifically, hyaluronic acid was modified to present pendant norbornene or β-cyclodextrin groups, and carefully controlled amounts of thiolated adamantane peptide and a di-thiol crosslinker were added to tune the number of covalent and supramolecular crosslinks present in the hydrogel post-photocrosslinking.

Although traditional dual network approaches employing photocrosslinking and Michael addition reactions successfully tune hydrogel properties, nonspecific chemical reagents can cross-react with surrounding tissues and encapsulated cargo, altering cellular phenotypes and macromolecule bioactivity. Bioorthogonal crosslinking strategies that promote gentler and more selective hydrogel formation and modification represent a promising alternative approach for forming dual networks in supramolecular hydrogels for sensitive ocular tissue engineering applications [28]. Reaction partners in bioorthogonal reactions do not occur in nature or cross-react with the common functional groups present in biology. Bioorthogonal chemistries have been extensively reviewed elsewhere, and interested readers are directed to several excellent recent reviews [28,95,96,97].

Bioorthogonal crosslinking has already shown promise for primary hydrogel network formation in ophthalmic tissue engineering, suggesting that these chemistries are compatible with the ocular surface and display reaction times suitable for both delivery and defect site retention. For example, our group recently reported the development of in situ forming corneal stromal substitutes based on collagen type I crosslinked by bio-orthogonal strain-promoted azide-alkyne cycloaddition (SPAAC), as well as SPAAC crosslinked hyaluronate–collagen hydrogels for suture-free corneal defect repair [22,23,24]. These bioorthogonally crosslinked hydrogels exhibited favorable optical properties and promoted healthy wound-healing in organ culture and in vivo rabbit corneal defect models. In addition to the SPAAC reaction, which employs readily available reaction pairs and has proven to be a powerful crosslinking strategy for the in situ formation of corneal stromal tissue replacements, recently reported biorthogonal reactions such as cucurbit[6]uril (CB[6])-promoted azide–alkyne cycloaddition and isonitrile–chloroxime ligation may act as useful alternative chemistries for forming dual networks in host–guest hydrogels. The small, cytocompatible reactive groups used in these reactions reduce aggregation concerns during synthesis and storage and enable orthogonal SPAAC reactions to be used for further hydrogel modifications (e.g., growth factor or peptide tethering) [98,99].

## 6. Fiber-Reinforced Composites for Enhanced Strength

Another challenge to improving the therapeutic potential of supramolecular hydrogels is the homogeneous microstructure of the materials. The native corneal structure is comprised of aligned collagen fibrils. Along with their mechanically stabilizing role, these collagen fibrils in the cornea influence homeostasis by providing spatial guidance to fibroblasts and keratocytes. Cultured corneal cells are also responsive to topographical cues, including substrate curvature and fibril size and orientation. Illustratively, Wu et al. reported that human corneal stromal stem cells (hCSSCs) cultured on aligned poly(ester urethane)urea with an average diameter of 165 nm secreted a dense collagen matrix containing collagen fibrils with regular interfibrillar spacing [100]. In contrast, hCSSCs grown on similar fibers of random orientation produced abundant but unorganized ECM. On aligned fibrous matrices, the hCSSCs also exhibited higher gene expression levels for keratocyte markers. Similarly, the upregulation of keratocyte expression markers has been observed for cells cultured on patterned substrates under physiological strain [101]. Moreover, curvature alone has been shown to induce human corneal stromal cell alignment, resulting in more organized nanostructures with aligned collagen fibrils and higher expression of proteoglycans such as keratocan, lumican, and decorin [102].

In addition to acting as topographical cues, embedded fibers have also been used to improve the mechanical properties of corneal stromal tissue substitutes [103,104,105,106]. Nanofibers can be prepared through various techniques, including self-assembly, natural product isolation, electrospinning, direct writing, and 3D printing [105,107]. In fiber-reinforced composites, mechanical loads are largely borne by embedded fibers, improving strength and suturability [106,108]. In an early example of this approach, Tonsomboon and Oyen reported substantially enhanced mechanical properties for alginate hydrogels with embedded electrospun gelatin fibers [104].

Fiber chemistry, size, and orientation all affect the physical properties of fiber-reinforced composites, as well as cellular responses to the embedded fibers [109,110]. In most fiber-reinforced composites, fibers improve mechanical robustness but reduce hydrogel transparency. Reduced transparency is associated with nonuniform interfibrillar spacing and random fiber orientation, which result in incident light scattering and impaired vision [106,111]. In addition, fiber feature sizes that are not significantly smaller than the shortest wavelength of visible light (400 nm) must match the refractive index of the hydrogel matrix to prevent reflection and refraction at the fiber–hydrogel interface [105]. In Tonsomboon and Oyen’s composite system, the choice of fiber crosslinking method significantly altered hydrogel transparency, with gels with more highly crosslinked and mechanically robust fibers also exhibiting greater opacity [104].

To better recapitulate native stromal structure and transparency, Kong et al. fabricated microfibrous poly(ε-caprolactone)-poly(ethylene glycol) grids by direct writing and infused the scaffolds with gelatin-methacryloyl [106]. In combination with appropriately supplemented serum-free media, these composite hydrogels maintained the keratocyte-like phenotype of rat limbal stromal stem cells and promoted keratocyte-specific gene and protein expression. Moreover, the fibrous hydrogels supported stromal matrix synthesis and regeneration in a rat intrastromal keratoplasty model.

Although fiber embedding is a promising approach for influencing cell fate and improving the mechanical properties of implanted corneal stromal tissue substitutes, the importance of alignment in modulating transparency and cellular phenotype complicates their application in injectable hydrogel systems, where the fibers may be damaged or distorted during extrusion. To avoid presenting damaged or disordered fibers that may promote a myofibroblastic phenotype and deposition of abnormal ECM, fibrous scaffolds could be deposited in defects layer-by-layer and then infused with an injectable host–guest hydrogel. However, this approach is more invasive than a purely injectable tissue substitute and may require suturing to maintain the desired engineered substrate organization, limiting its application in high-risk patients.

## 7. Secreted Protein Capture for Directed Tissue Remodeling

Like their allogenic donor tissue counterparts, engineered tissue substitutes have been associated with high rates of graft failure to date [3], marking another significant challenge to widespread clinical use. Corneal tissue repair depends on the synthesis and assembly of organized extracellular matrix (ECM) components that recapitulate the mechanical strength and transparency of native tissue [6]. However, corneal injury induces keratocyte transformation into a myofibroblastic cell type that deposits atypical extracellular matrix in the healing defect [6,10]. An ideal corneal tissue substitute should successfully integrate with surrounding tissue and induce tissue-specific, healthy ECM synthesis to promote functional tissue regeneration.

While extrinsic signals regulate cell fate, cell-mediated remodeling and protein secretion can mask cues presented in engineered materials [29,112,113,114]. For example, mesenchymal stromal cells rapidly produce a pericellular extracellular matrix, with secreted proteins such as fibronectin detected within 24 h post-encapsulation [57,112]. Cells adhere to nascent proteins secreted in both covalently crosslinked and supramolecular hydrogels, with hydrogel crosslinking mediating the distribution and assembly of secreted proteins [57,114]. This bi-directional signaling between cells and their surrounding environment influences mechanosensing and cell fate decisions [57].

Although secreted ECM complicates the design of hydrogel-based corneal stromal tissue substitutes, the dynamic reciprocity observed between cells and their microenvironment could be harnessed to regenerate complex corneal tissue. Specifically, cell-mediated structural anisotropy would better recapitulate the healthy tissue microstructure that facilitates the cornea’s unique functional properties. For example, the highly organized collagen lamellae in the native corneal stroma enable tissue transparency due to their small (~25 nm) diameter, regular spacing, and tight packing [3], an organization that is challenging to directly recapitulate in engineered injectable materials.

**Figure 8:**
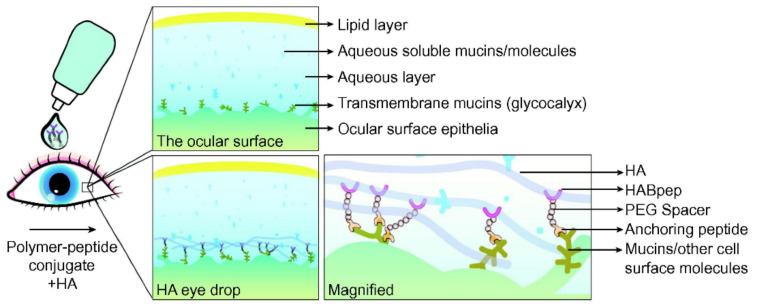
Idealized schematic showing targeted delivery of hyaluronic acid to the ocular surface. Targeted delivery is achieved using a polymer-peptide conjugate containing a sialic-acid-binding anchoring peptide and a hyaluronic-acid-binding peptide (HABpep) linked by a PEG spacer. Reproduced with permission. Copyright 2017, Elsevier [121].

To create a multilayered matrix reminiscent of the native stromal ECM and promote functional recovery, endogenous or transplanted cells could be encouraged to secrete specific ECM molecules by incorporating ECM-regulating growth factors in the hydrogel network [113]. Alternatively, ECM capture peptides or aptamers may be tethered in the hydrogel, increasing local retention of desired macromolecules and improving integration with surrounding tissues [113,115]. While excess host macrocycles in host–guest hydrogels already aid integration through non-specific interactions with aromatic amino acids in native tissue [60,116,117], ECM capture peptides may enable specific targeting and spatiotemporally controlled hydrogel remodeling. For example, Cooper-White and colleagues have deployed protein binding peptides in 3D PEG hydrogels to retain desired ECM proteins, including fibronectin, laminin, and type I collagen [118]. Similarly, Elisseeff and coworkers identified a hyaluronic acid binding peptide that, when conjugated to eight-arm PEG, exhibited tissue specific retention, enabling targeting to damaged cartilage with exposed hyaluronic acid [119]. Moreover, Elisseeff and coworkers have reported the anchoring of a polymer–peptide system containing both a hyaluronic acid binding peptide and a collagen type I binding peptide; the collagen-binding peptide exhibited non-specific binding to damaged corneal, scleral, and conjunctiva tissue, while the hyaluronic-acid-binding peptide recruited and retained lubricating hyaluronic acid at the ocular surface [120]. Similarly, a sialic-acid-binding peptide has been employed in targeted delivery of hyaluronic acid to the ocular surface epithelium (Figure 8) [121].

A summary of ECM capture peptides validated in hydrogel biomaterial and targeted drug-delivery applications is provided in Table 2.

**Figure 9:**
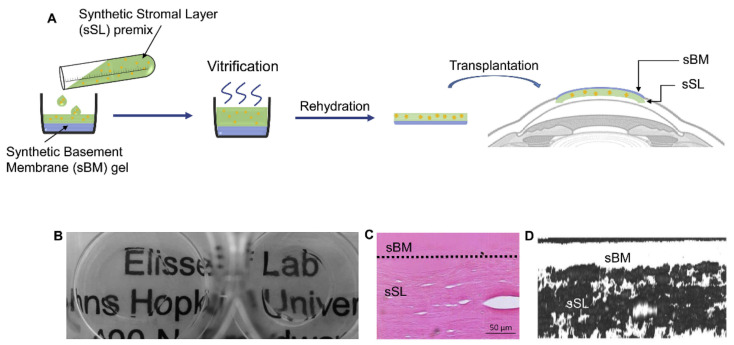
A dual-layered corneal tissue substitute comprising a synthetic stromal layer and synthetic basement membrane gel facilitated rapid re-epithelialization and exhibited favorable optical and biological properties. (**A**) Idealized schematic of hydrogel preparation and transplantation process. (**B**) Photograph demonstrating hydrogel transparency. (**C**) H&E and (**D**) second harmonic generation imaging of a cross-section of the dual-layered construct showing different microstructures within the two gel layers. Reproduced with permission. Copyright 2020, Elsevier [128].

To promote corneal reconstruction, different ECM capture peptides could be incorporated in a layer-by-layer fashion reminiscent of healthy ocular tissue (e.g., through sequential injection of host or guest functionalized polymers further modified with different ECM capture peptides or by mixing or diffusing different guest-tagged ECM capture peptides into each layer). For example, the corneal stroma includes highly organized type I and V collagen, as well as fibril-associated collagens XII and XIV and nonfibrillar type VI collagen [3]. In the stroma, type I collagen is the major structural component of collagen fibrils, while collagen type V regulates fibril size and assembly [10]. Other layers of the cornea possess different amounts and distributions of collagen and proteoglycans that facilitate their unique functions. For example, Descemet’s membrane, which supports the corneal endothelium beneath the stroma, is a basement membrane rich in type VIII collagen, while Bowman’s layer underneath the epithelium is primarily composed of dense, randomly oriented type I and V collagen [3,127]. Multi-layered tissue substitutes that recapitulate some of the complexity of native corneal ECM have recently been reported. Illustratively, in 2020, Elisseeff and colleagues described an engineered, dual-layered system comprising a synthetic Bowman’s membrane and a synthetic stromal layer to facilitate rapid re-epithelialization and to provide mechanical support and modulate inflammation, respectively (Figure 9) [128].

In addition to recapitulating the multilayered ECM organization in the healthy cornea, ECM capture peptides could be used to encourage more rapid reinnervation and reepithelialization post-injection. For example, laminin capture peptides may prove useful for promoting corneal nerve regeneration, a current bottleneck in functional recovery. Grafted laminin-derived YIGSR peptides have been reported to promote corneal epithelial stratification and nerve regeneration in vitro [129], an effect that may be enhanced by enrichment of the full-length protein. Moreover, hyaluronic acid has shown promise in promoting corneal reepithelialization following injury [130,131,132]. By increasing retention of the hyaluronic acid secreted by corneal epithelial cells during the wound-healing process [133], recovery may be accelerated without the repeated topical application of hyaluronic-acid-containing eye drops.

## 8. Conclusions

The transparent, avascular cornea serves as the main refractive surface of the visual system, contributing about two-thirds of the eye’s refractive power while protecting intraocular structures from environmental threats. Corneal damage due to trauma or disease can be sight-threatening, and the need for replacement corneal tissue vastly outstrips the number of donor corneas that are available worldwide. The field of corneal tissue engineering has arisen to address the need for readily available tissue substitutes that can precisely fill corneal defects in a minimally invasive manner. Among injectable materials, host–guest hydrogels show great potential for corneal reconstruction due to their tunability and selectivity. In these hydrogels, molecular recognition interactions between macrocyclic host molecules and small molecule guests create physically crosslinked networks capable of rapid shear-thinning and self-healing upon stress application. Post-injection, host–guest hydrogels homogenously distribute encapsulated payloads and conform to defect site geometry, reducing the risk of visual disturbance due to poor wound apposition and rough surface distortions. To fully realize the promise of host–guest hydrogels as a corneal tissue substitute, secondary crosslinked networks and embedded fibers can be employed to improve gel strength and stability. Additionally, ECM capture peptides associated with host–guest hydrogels may promote scaffold remodeling into healthy, multilayered tissue. Ultimately, corneal tissue substitutes exploiting host–guest interactions may permit the scalable, minimally invasive replacement of damaged cornea tissue, transforming the standard of care for patients suffering from cornea-related blindness.

## Figures and Tables

**Table 1:** Host–guest pairs employed in hydrogel biomaterials and their respective binding affinities.

Host	Guest(s)	Keq	Ref
β-cyclodextrin	adamantane	10^4^–10^5^ M^−1^	[46]
	aromatic amino acids	10^1^–10^2^ M^−1^	[47]
cucurbit[6]uril	1,6-diaminohexane	10^6^–10^8^ M^−1^	[48]
cucurbit[7]uril	phenylalanine	10^7^ M^−1^	[49]
	ferrocene	10^8^ M^−1^	[49]
	*p*-xylylenediamine	10^9^ M^−1^	[49]
	adamantane	10^10^–10^12^ M^−1^	[49]
cucurbit[8]uril	viologen and naphthoxy	10^11^–10^12^ M^−2^	[50]
	Phe-Gly-Gly	10^11^ M^−2^	[48]

**Table 2:** ECM capture peptides validated in hydrogel biomaterial and targeted drug delivery applications.

ECM Molecule	Capture Peptide Sequence	References
Collagen I	GLRSKSKKFRRPDIQYPDATDEDITSHM	[118,122]
Collagen I (decorin-derived)	SYIRIADTNIT	[121,123]
Collagen II	WYRGRL	[120]
Fibronectin	GGWSHW	[118,124]
Hyaluronic acid	STMMSRSHKTRSHHV	[119,121,125]
Hyaluronic acid	GAHWQFNALTVR	[120]
Laminin	IPCNNKGAHSVGLMWWMLAR	[118,126]
Sialic acid	GGSPYGRC	[121]

## Data Availability

Not applicable.
